# Pioglitazone Metformin Complex Improves Polycystic Ovary Syndrome Comorbid Psychological Distress via Inhibiting NLRP3 Inflammasome Activation: A Prospective Clinical Study

**DOI:** 10.1155/2020/3050487

**Published:** 2020-04-28

**Authors:** Qing-jun Guo, Jing Shan, Yi-feng Xu, Yan-yan Hu, Cui-lan Huo, Jing-yun Song, Chao-qun Wang, Hui Zhou, Chao-qin Yu, Qin Huang

**Affiliations:** ^1^Department of Endocrinology, Changhai Hospital, Naval Medical University, Shanghai, China; ^2^Department of Traditional Chinese Medicine, Changhai Hospital, Naval Medical University, Shanghai, China; ^3^Changning Retirement Cadre Rest Care Clinic, Garrison District Shanghai, China

## Abstract

**Objective:**

This study aimed at investigating the therapeutic effect and mechanism of pioglitazone metformin complex preparation (PM) in polycystic ovary syndrome (PCOS) comorbid psychological distress.

**Methods:**

Seventy-five patients with PCOS comorbid psychological distress were randomly allocated into the PM, metformin, and placebo groups. The primary efficacy measure was the change from baseline to week 12 on the Symptom Checklist 90-R (SCL-90-R) scores. NLRP3 inflammasome, IL-1*β*, IL-6, TNF-*α*, and biochemical parameters were determined at baseline and at week 12. The participants were required to meet the criteria for PCOS (Rotterdam, NIH) and psychological distress (any factor scores of SCL − 90 − R > 2).

**Results:**

The participants had significantly high scores on the SCL-90-R scales of anxiety and depression. PM significantly decreased anxiety and depression symptom severity (from 2.31 ± 0.75 to 1.65 ± 0.38, *p* < 0.001, and from 2.08 ± 0.74 to 1.61 ± 0.46, *p* = 0.010, at week 12, respectively). PM significantly decreased the expression of NRPL3 and caspase-1. Patients in the PM group experienced a significant reduction in IL-1*β* (from 98.42 ± 14.38 to 71.76 ± 13.66, *p* = 0.02), IL-6 (from 87.51 ± 8.74 to 71.98 ± 15.87, *p* = 0.02), and TNF-*α* (from 395.33 ± 88.55 to 281.98 ± 85.69, *p* = 0.04). PM was superior to metformin in reducing total testosterone (2.24 ± 0.74 versus 3.06 ± 0.83, *p* = 0.024, at week 12).

**Conclusions:**

This study is the first to reveal that PM alleviates psychological distress via inhibiting NLRP3 inflammasome and improves several markers, including total testosterone.

## 1. Introduction

Polycystic ovary syndrome (PCOS) is a heterogeneous disease with an incidence rate of about 6%–10% in women of reproductive age [1]. The clinical manifestations of PCOS include hirsutism, acne, acanthosis, obesity, and infertility, which are associated with great psychological burden among patients. Previous studies have reported that women with PCOS have a low quality of life and undergo immense pressure due to various clinical symptoms and changes in body functions [2, 3]. Long-term anxiety and increasing stress levels are important factors in the pathophysiology of psychological distress. The prevalence of psychological distress in patients with PCOS is as high as 30%–57% [4]. However, the relationship between PCOS and psychological distress is complex, and its mechanism remains unclear [5]. In addition, no clinical studies have been reported. Therefore, the mechanisms must be elucidated to develop treatment strategies for PCOS comorbid psychological distress.

Metformin and pioglitazone have been reported in the treatment of PCOS. These drugs exert a synergistic therapeutic effect on the treatment of type 2 diabetes. Several studies have reported the anti-inflammatory and antidepressant effects of pioglitazone on psychiatric conditions [6–8]. Furthermore, animal studies demonstrated that a combination of metformin and pioglitazone exerts a better effect on ameliorating PCOS than when used individually [9]. However, clinical research on the use of pioglitazone metformin complex preparation (PM) in the treatment of PCOS comorbid psychological distress is lacking.

In recent years, alterations in the regulation of immunity, especially the inflammatory response system, have been associated with the occurrence of psychological distress [10, 11]. Su et al. found that the expression levels of IL-1*β* in the serum and the hippocampus significantly increase in a mouse model experiencing chronic stress. This result suggests that chronic inflammation is involved in the occurrence of psychological distress [12]. NLRP3 inflammasome plays an important role in this process, and it is the key driver of neuroinflammation [13]. NLRP3 through an apoptosis-associated speck-like protein containing a CARD (ASC), caspase-1, constitutes NLRP3 inflammasome, which in turn induces the maturation and release of IL-1*β*, IL-33, and other related inflammatory mediators. This phenomenon triggers inflammatory reactions and disease development and progression. Surprisingly, previous studies showed that NLRP3 inflammasome is activated in various diseases, such as metabolic syndrome, Alzheimer's disease, and autoimmune diseases [14]. Majority of these diseases have been associated with high incidence rates of psychological distress. Other clinical studies have found that chronic inflammatory factors, such as ILs, TNF-*α*, PAI-1, and MCF-1, are increased in the peripheral blood of patients with PCOS to varying degrees [15].

On the basis, our study aimed at investigating the therapeutic effect and mechanism of PM in PCOS comorbid psychological distress.

## 2. Materials and Methods

### 2.1. Trial Setting and Design

This prospective clinical study was approved by the Changhai Hospital Medical Ethics Committee (approval number: CHEC2015-135). All study participants were presented with an informed consent, which they were required to read carefully, sign, and return a copy to the researcher prior to the study. This was also registered on the China Clinical Trials Registry website (Registration No.: ChiCTR-OPC-17011930).

### 2.2. Participants

The participants attending the outpatient clinic of the Changhai Hospital in Shanghai from October 2016 to July 2018 were considered for the study. The inclusion criteria were as follows: (1) aged between 20 and 35 years old; (2) met the PCOS diagnostic criteria (Rotterdam, NIH): (a) ultrasound examination revealed polycystic ovary, at least unilateral ovaries with follicles of 2–9 mm in diameter and/or ovarian volume ≥ 10 mL; (b) clinical and/or biochemical presence of excessive androgen; and (c) rare or no ovulation, in accordance with any two of the above three items; and (3) met the diagnostic criteria for psychological distress based on the Symptom Checklist 90-R (SCL-90-R): any factor scores of SCL − 90 − R > 2.

The exclusion criteria were as follows: (1) Patients had diseases that cause androgen excess and nonovulation, such as ovarian or adrenal tumors, Cushing's syndrome, congenital adrenal hyperplasia, hypothyroidism, and hyperprolactinemia; (2) The patients had a history of treatment with antidiabetic drugs, hypolipidemic drugs, and hormones within the past 6 months prior to the commencement of the study; (3) The patients had a history of major life events within 6 months; (4) Apart from PCOS, the patient had other serious physical illnesses; and (5) Patients who had low education levels and could not comprehend the meaning of the table.

The sample size in phase 2 study was calculated as 96 cases (*N* = 96). Sample size was determined from the calculation formula below.

(1) $$\begin{array}{c} \begin{array}{c} N=Z^{2}\times \left(\text{Pp}\times \left(1-\text{Pp}\right)\right)/E^{2},\\ N=\text{sample size estimate},\\ Z\left(\text{statistics}\right)=1.96\left(\text{confidence interval}=95\%\right),\\ E\left(\text{error values}\right)=10\%,\\ p\left(\text{probability values}\right)=0.5. \end{array} \end{array}$$

### 2.3. Administration of Drugs and Randomization

Patients with PCOS comorbid psychological distress were randomly assigned to three treatment groups: placebo group (placebo, 3 tablets/day), metformin group (M, metformin 500 mg/tablet, 3 tablets/day), and PM group (PM, each tablet containing 15 mg of pioglitazone and 500 mg of metformin, 3 tablets/day). The total course of treatment was 12 weeks. All pills were identical in taste and shape. A computer-generated random code was used to assign random numbers to the patients. The patients were blinded to the administration of the drugs. The drugs (metformin, placebo, and pioglitazone metformin complex preparation) were provided free of charge by Hangzhou Zhongmei Huadong Pharmaceutical Co., Ltd.

### 2.4. Outcome Measures

SCL-90-R, a self-report questionnaire of 90 items on a 5-point Likert scale (ranging from “1 = no problem” to “5 = very serious”), is a good tool for assessing multidimensional psychopathological aspects (9 factors: depression, anxiety, obsessive-compulsive, somatization, hostility, interpersonal sensitivity, paranoid ideation, phobic anxiety, and psychoticism) [16]. SCL-90-R has good reliability and validity in the Chinese population [17]. In general, compared with a normal Chinese, there are 3 ways to identify positive psychological symptoms of SCL-90-R: the total score is greater than 160; out of 90 items, the number of positive items (scores > 2) is greater than 43; any of 9 factors has a score greater than 2. Based on our previous results, any factor scores of SCL − 90 > 2 was defined as criterion.

The body mass index (BMI), inflammation, metabolic, and hormonal parameters were measured at baseline and at week 12. On the third day of menstruation or amenorrhea (menstrual cessation for more than 6 months and B-ultrasound prompt no dominant follicle), the venous blood of the study participants was taken on fasting to measure the levels of luteinizing hormone (LH), follicle-stimulating hormone (FSH), prolactin (PRL), total testosterone (TT), fasting blood glucose, and insulin release. HOMA-IR was calculated as fasting glucose × fasting insulin/22.5.

The levels of IL-1*β*, IL-6, and TNF-*α* in serum were measured by ELISA.

### 2.5. Serum and Human Peripheral Blood Mononuclear Cell (PBMC) Extraction

At baseline and at week 12, the participants fasted the previous night and their blood was drawn intravenously between 8 and 10 am the following morning. The blood sample was centrifuged, the supernatant pipetted, dispensed into an EP tube, and stored at -80°C until use. PBMC was separated from heparinized blood by density gradient centrifugation using the Ficoll lymphocyte separation solution (Solarbio, China) [18].

### 2.6. Real-Time Quantitative PCR Analysis of the mRNA Expression Levels of NLRP3 and Caspase-1

The mRNA expression levels of NLRP3 and caspase-1 in PBMC were detected by SYBR Green quantitative PCR. Total RNA extraction was performed by TRIzol lysis, and RNA quality and concentration were measured using a nucleic acid-protein quantitative detector (Eppendorf). The extracted RNA was reverse transcribed into cDNA using the Reverse Transcription Kit (Takara, Japan). The reverse transcription conditions were set as follows: 37°C for 15 min; 85°C for 5 s; NLRP3 primer sequence: 5′-GGAGAGACCTTTATGAGAAAGCAA-3′ (forward) and 5′-GCTGTCTTCCTG GCATATCACA-3′ (reverse); and caspase-1 primer sequence: 5′-CCGAAGGTGATCATCATCCA-3′ (forward) and 5′-ATAGCATCATCCTCAAACTCTTCTG-3′ (reverse). *β*-Actin was used as an internal reference, and the primer sequences were 5′-CCAGATCATGTTTGAGACC-3′ (forward) and 5′-ATGTCACGCACGATTTCCC-3′ (reverse). The thermal cycling conditions were set as follows: Step 1: 95°C, 30 s, predenaturation; Step 2: 95°C, 5 s; and Step 3: 60°C, 30 s, 40 cycles of amplification. The second step of each cycle was a collection of the fluorescent signals. In this study, the relative expression of the target and reference genes was calculated using the 2^-*ΔΔ*Ct^ method.

### 2.7. Statistical Analysis

Data were entered and statistically processed using the SPSS 22.0 statistical software. The measurement data were expressed as mean ± standard deviation ($\bar{\text{X}}\pm \text{S}$), and the comparison between groups was analyzed by the *t*-test or one-way ANOVA (GraphPad Software, Inc., CA, USA). *p* < 0.05 was considered to indicate statistical significance.

## 3. Results

### 3.1. Participants

This study recruited 111 patients; 35 patients were enrolled in the placebo group but 14 patients dropped out of the study, 35 patients were enrolled in the M group but 9 patients dropped out, and 41 patients were enrolled in the PM group but 13 patients dropped out. The final number of participants in this phase was 75 patients, where in placebo group, *n* = 21; M group, *n* = 26; and PM group, *n* = 28.

The patients dropped out of the study for several reasons. The main reason was that 14 patients had accidental pregnancy (2 patients in the placebo group, 4 in the M group, and 8 in the PM group). Other reasons were poor compliance, busy working, and so on. The total dropping out rate was 31.53%, whereas after elimination of unintended pregnancy, it was 19.82%. The long duration also affected drop-out rates. The results revealed no differences in baseline data among the three groups (Table 1).

### 3.2. Effects of PM on PCOS Comorbid Psychological Distress

The participants showed a high score in two scales: depression and anxiety (Table 2). Metformin and placebo showed no improvement on symptoms of psychological distress, whereas PM significantly reduced symptoms of depression and anxiety (*F* = 5.698, *p* = 0.008, and *F* = 6.391, *p* < 0.001, respectively, week 12). Self-reported assessments of anxiety symptom severity reflected an improvement with mean SCL-90-R scores decreasing from 2.31 ± 0.75 at baseline to 1.65 ± 0.38, *p* < 0.001. Depression symptoms decreased significantly from a mean of 2.08 ± 0.74 to 1.61 ± 0.46, *p* = 0.010.

### 3.3. NLRP3 Inflammasome Activation Was Inhibited by PM

**Figure 1:**
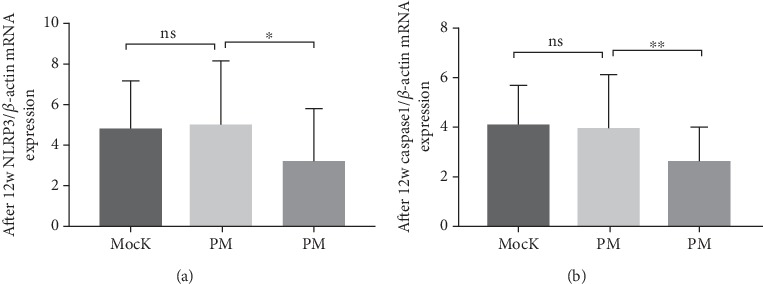
PM regulates the gene expression of NRPL3 and caspase-1. qRT-PCR analysis indicated that NRPL3 (a) and caspase-1 (b) were significantly downregulated in patients with PCOS comorbid psychological distress. ^∗^*p* < 0.05, ^∗∗^*p* < 0.01 by Student's *t*-test.

NLRP3 inflammasome is closely related to neuroinflammation [19]. The preliminary research of our team found that NLRP3 inflammasome was activated in patients with PCOS comorbid psychological distress compared with patients with PCOS (Figure S1). To assess whether PM could inhibit NLRP3 inflammasome activation, we assessed the effect of PM on NLRP3 inflammasome and IL-1*β* secretion. PM reduced the expression of NLPR3 (Figure 1(a), *F* = 3.374, *p* = 0.040). Moreover, PM treatment suppressed caspase-1 activation (Figure 1(b), *t* = 6.011, *p* = 0.004) and IL-1*β*production (Table 3, *F* = 3.612, *p* = 0.032). Metformin showed no effect on the inhibition of NLRP3 inflammasome.

### 3.4. Effect of Treatment on Inflammatory Parameters

No significant difference in inflammatory parameters was found between the placebo and M groups, but PM significantly decreased the levels of IL-1*β*, IL-6, and TNF-*α* (*F* = 3.612, *p* = 0.032; *F* = 6.124, *p* = 0.003; and *F* = 6.191, *p* = 0.003, respectively, week 12) (Table 3). Patients in the PM group experienced significant reductions in IL-1*β* (from 98.42 ± 14.38 to 71.76 ± 13.66, *p* = 0.02), IL-6 (from 87.51 ± 8.74 to 71.98 ± 15.87, *p* = 0.02), and TNF-*α* (from 395.33 ± 88.55 to 281.98 ± 85.69, *p* = 0.04).

### 3.5. Effect of Treatment on Biochemical and Hormonal Profiles

The results showed no changes in LH, FSH, and PRL among the three groups (Table 4). No significant difference in BMI and HOMA-IR was detected among the three groups. However, TT significantly reduced in the PM group (*F* = 10.180, *p* < 0.001, at week 12). PM was more effective and better than metformin alone in reducing testosterone levels (*p* = 0.024).

## 4. Discussion

To the best of our knowledge, this is the first prospective study exclusively investigating the effects of PM in patients with PCOS comorbid psychological distress. Patients with PCOS showed several symptoms of anxiety and depression. PM can significantly improve anxiety and depression symptom severity. In addition, PM can effectively inhibit the activation of NLRP3 inflammasome and decrease the release of proinflammatory cytokines.

Unfortunately, a special medicine for patients with PCOS comorbid psychological distress is lacking. PCOS is a multifactorial disease, and its treatment focuses on lifestyle management. The current drug treatments of PCOS mainly contain oral contraceptive pills and insulin-sensitizing drugs, which have no antidepressant effect [20]. Pioglitazone, a member of thiazolidinediones, has recently been reported to be a potential drug for treatment of depression because of its anti-inflammatory effects [21, 22]. Therefore, we suggested that the combination of metformin and pioglitazone is suitable for such patients. The results show that PM has advantages over metformin alone, which is in line with our previous findings.

Our results are in consistent with animal models of depression with NLRP3 inflammasome activation [23, 24]. Microtubule-affinity regulating kinase 4, migration inhibitory factor, thioredoxin-interacting protein, and Nek7 regulate the activation of NLRP3 inflammasome [14]. The inflammasome is a multiprotein intracellular complex that detects harmful stimuli and induces caspase-1 activation, leading to the secretion of inflammatory cytokines, such as IL-1*β* [10]. IL-1*β* may drive the induction of indolamine-2, 3-dioxygenase, which catabolizes tryptophan into kynurenine and thereby reduces the available pool of tryptophan for 5-HT synthesis [25]. Then, the decreased 5-HT may mediate the onset of mood disorders [26]. Increasing evidence suggests that inflammation participates extensively in depression [27, 28]. Inflammatory mediators affect the brain through saturable transportation [29] or cerebral endothelial [30]. The hypothalamic–pituitary–adrenal axis could synthesize inflammatory mediators (e.g., IL-1, IL-6, and TNF-*α*) during psychological distress [31, 32]. Proinflammatory cytokines, including IL-1*β*, IL-6, and TNF-*α*, are elevated in experimental and clinical studies of depression [33–35]. In addition, TNF-*α* is correlated with depression severity [36]. Thus, NLRP3 inflammasome and inflammation play important roles in psychological distress. However, the mechanism by which PM inhibits the activation of NLRP3 inflammasome warrants further study.

PM was more effective and better than metformin alone in reducing testosterone levels. However, the current research failed to clarify the relationship between testosterone and psychological distress in patients with PCOS. A meta-analysis reported that psychological distress is weakly associated with BMI, insulin resistance, and elevated testosterone [20]. Borghi et al. found an inverse relationship between levels of testosterone and trait anger in patients with PCOS [37], which is consistent with the results of Ehrmann et al. [38]. Roepke et al. reported that levels of testosterone in patients with borderline personality disorder are positively related to depression scores [39].

## 5. Limitations

This study has some limitations. According to clinical manifestations, PCOS is divided into different subtypes, such as obese and nonobese. The degree of an emotional disorder is also graded. However, the sample size in the present study was very small. In addition, the observation period was long, and the drop-out rate was high. Therefore, not all the above factors were considered. Longer interventions with larger sample sizes are needed in future studies to confirm the current study findings. Moreover, PM, as a new composite preparation, has no clinical evidence for pregnancy safety. However, our research group will continue to conduct follow-up studies on its safety.

## 6. Conclusions

In summary, this clinical research is the first to investigate the effect of PM complex preparation in patients with PCOS comorbid psychological distress. PM improved the inflammation, inhibited the activation of NLRP3 inflammasome, and reduced the release of IL-1*β*.

## Figures and Tables

**Table 1:** Baseline data.

	Placebo (*n* = 21)^a^	M (*n* = 26)^a^	PM (*n* = 28)^a^
Age	26.9 ± 4.5	27.4 ± 4.1	26.1 ± 4.98
BMI	24.00 ± 4.82	23.54 ± 3.34	24.00 ± 4.10
Marital status, *n* (%)	Single: 7 (33.3%), married: 13 (61.9%), divorced: 1 (4.8%)	Single: 8 (30.7%), married: 15 (57.7%), divorced: 3 (11.6%)	Single: 8 (28.6%), married: 18 (64.3%), divorced: 2 (7.1%)
Level of education, *n* (%)	Under high school: 1 (4.7%), diploma: 1 (4.7%), higher diploma: 19 (90.6%)	Under high school: 1 (3.8%), diploma: 1 (3.8%), higher diploma: 24 (92.4%)	Under high school: 1 (3.5%), diploma: 1 (3.5%), higher diploma: 36 (93.0%)

BMI: body mass index; PCOS: polycystic ovary syndrome. ^a^Placebo group represents patients given placebo. M group represents patients given metformin. PM group represents patients given pioglitazone metformin complex preparation.

**Table 2:** SCL-90-R scores at baseline and week 12.

Psychological distress (SCL-90-R)	Placebo	M	PM	*p*	Placebo	M	PM	*p*
	Baseline				Week 12			
Somatization	1.69 ± 0.58	1.76 ± 0.65	1.70 ± 0.53	0.144	1.71 ± 0.56	1.73 ± 0.51	1.50 ± 0.36	0.210
Obsessive-compulsive	1.79 ± 0.54	1.93 ± 0.75	1.89 ± 0.73	0.567	1.75 ± 0.62	1.80 ± 0.54	1.66 ± 0.50	0.378
Interpersonal sensitivity	1.56 ± 0.56	1.66 ± 0.60	1.53 ± 0.54	0.456	1.63 ± 0.42	1.56 ± 0.49	1.47 ± 0.39	0.498
Depression	2.12 ± 0.72	2.14 ± 0.65	2.08 ± 0.74	0.71	2.16 ± 0.68	2.04 ± 0.60	1.61 ± 0.46	0.008
Anxiety	2.34 ± 0.55	2.26 ± 0.67	2.31 ± 0.75	0.579	2.35 ± 0.53	2.20 ± 0.69	1.65 ± 0.38	<0.001
Hostility	1.71 ± 0.70	1.94 ± 0.77	1.72 ± 0.68	0.476	1.72 ± 0.67	1.74 ± 0.62	1.55 ± 0.52	0.279
Phobic anxiety	1.59 ± 0.36	1.59 ± 0.70	1.41 ± 0.53	0.813	1.66 ± 0.50	1.38 ± 0.36	1.44 ± 0.36	0.525
Paranoid ideation	1.69 ± 0.75	1.58 ± 0.62	1.50 ± 0.68	0.721	1.82 ± 0.88	1.65 ± 0.68	1.49 ± 0.42	0.691
Psychoticism	1.58 ± 0.47	1.67 ± 0.68	1.60 ± 0.67	0.543	1.83 ± 0.68	1.56 ± 0.45	1.49 ± 0.40	0.443

**Table 3:** Inflammatory cytokine levels at baseline and week 12.

Variations	Placebo	M	PM	*p*	Placebo	M	PM	*p*
	Baseline				Week 12			
IL-1*β* (pg/mL)	98.64 ± 9.58	99.60 ± 7.91	98.42 ± 14.38	0.92	95.96 ± 14.95	96.44 ± 14.38	71.76 ± 13.66	0.032
IL-6 (pg/mL)	86.72 ± 9.97	85.23 ± 10.31	87.51 ± 8.74	0.674	88.47 ± 14.98	86.43 ± 23.08	71.98 ± 15.87	0.003
TNF-*α* (pg/mL)	395.73 ± 78.22	414.97 ± 77.78	395.33 ± 88.55	0.35	386.45 ± 89.06	408.22 ± 47.78	281.98 ± 85.69	0.003

**Table 4:** Clinical features and biochemical parameters at baseline and week 12.

Variations	Placebo	M	PM	*p*	Placebo	M	PM	*p*
	Baseline				Week 12			
BMI (kg/m^2^)	24.00 ± 4.82	23.54 ± 3.34	24.00 ± 4.10	0.898	23.88 ± 3.95	21.90 ± 3.05	22.48 ± 3.16	0.13
LH (IU/L)	9.72 ± 2.23	9.61 ± 3.74	9.53 ± 3.31	0.992	9.82 ± 1.80	7.20 ± 1.80	6.33 ± 1.98	0.087
FSH (IU/L)	6.28 ± 2.10	6.31 ± 1.52	6.17 ± 1.70	0.962	6.26 ± 1.56	6.46 ± 1.37	5.32 ± 1.03	0.447
PRL (*μ*g/L)	13.54 ± 2.17	13.69 ± 2.27	13.51 ± 2.97	0.995	13.16 ± 2.80	13.69 ± 2.82	14.50 ± 2.57	0.448
TT (*μ*g/L)	0.77 ± 0.22	0.82 ± 0.22	0.77 ± 0.23	0.876	0.67 ± 0.24	0.60 ± 0.19	0.48 ± 0.13^∗^	<0.001
HOMA-IR	3.21 ± 1.30	3.41 ± 0.65	3.20 ± 0.53	0.861	3.28 ± 1.42	3.06 ± 0.83	2.24 ± 0.74	0.076

^∗^Group PM vs. group M at 12 w; *p* = 0.024.

## Data Availability

The data used to support the findings of this study have not been made available.

## Supplementary Materials

Supplementary Materials Figure S1: expression levels of NLRP3 and caspase-1 in PBMC in patients with PCOS comorbid psychological distress (PPD). (A, B) Gene expressions of NLRP3 and caspase-1 as determined by qRT-PCR (control group, *n* = 30; PCOS group, *n* = 38; and PPD group, *n* = 40). (C) Western blot analysis to detect NLRP3 protein expression in PBMC (control group, *n* = 3; PCOS group, *n* = 3; and PPD group, *n* = 3). (D) Gray value of the Western blot results. ^∗∗^*p* < 0.01 and ^∗∗∗∗^*p* < 0.0001 compared with the control group. ^#^*p* < 0.05 and ^####^*p* < 0.0001 compared with the PCOS group. The result is representative of three biological replicates.
