# Supplementary material for: Pioglitazone Metformin Complex Improves Polycystic Ovary Syndrome Comorbid Psychological Distress via Inhibiting NLRP3 Inflammasome Activation: A Prospective Clinical Study
Source: Mediators Inflamm. 2020 Apr 28;2020:3050487. doi: 10.1155/2020/3050487 (PMC7204303; doi:10.1155/2020/3050487)

Supplementary Fig. 1 Expression levels of NLRP3 and caspase-1 in PBMC in patients with PCOS comorbid psychological distress (PPD)

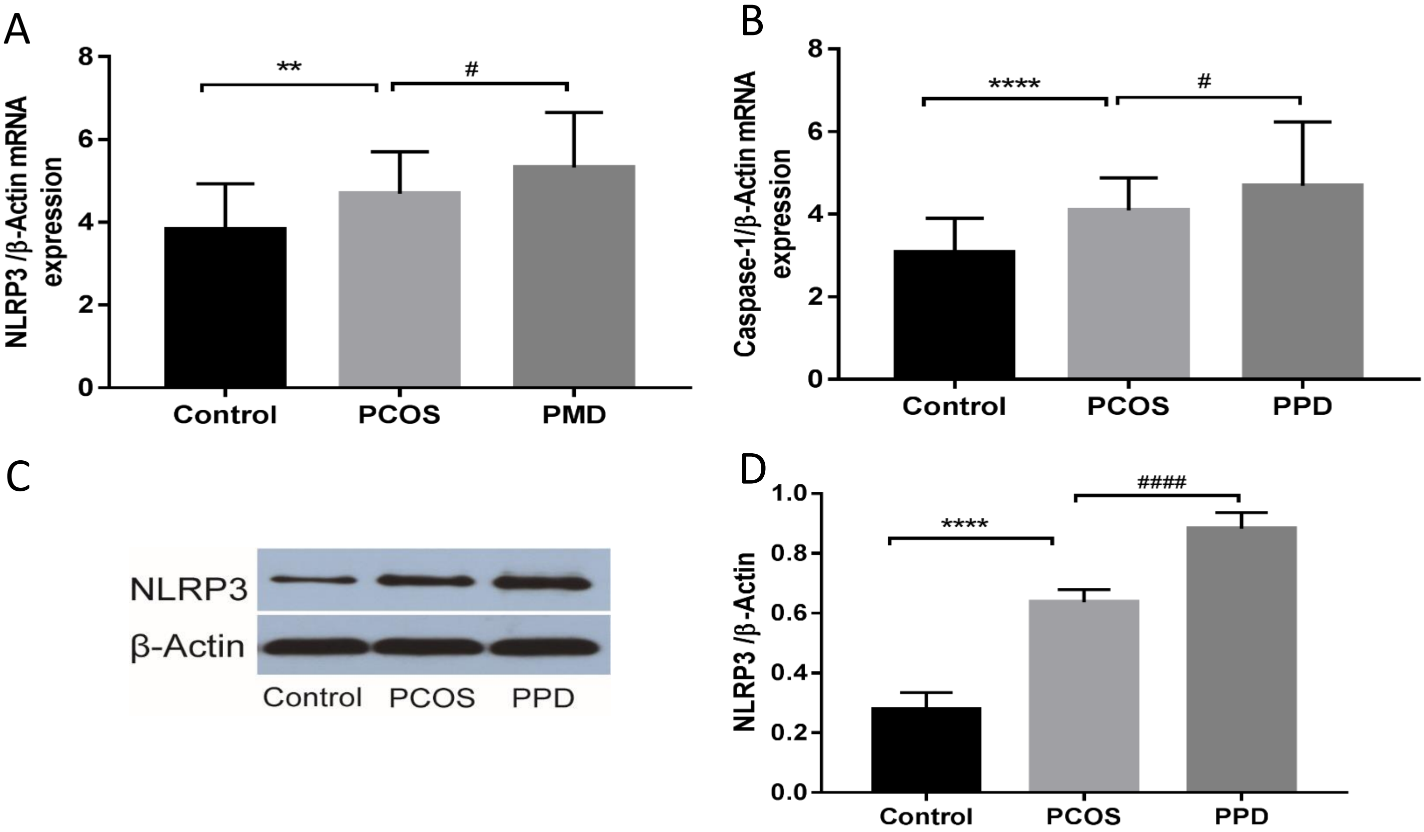

Supplement: Supplementary Materials — Figure S1: expression levels of NLRP3 and caspase-1 in PBMC in patients with PCOS comorbid psychological distress (PPD). (A, B) Gene expressions of NLRP3 and caspase-1 as determined by qRT-PCR (control group, n = 30; PCOS group, n = 38; and PPD group, n = 40). (C) Western blot analysis to detect NLRP3 protein expression in PBMC (control group, n = 3; PCOS group, n = 3; and PPD group, n = 3). (D) Gray value of the Western blot results. ∗∗p < 0.01 and ∗∗∗∗p < 0.0001 compared with the control group. #p < 0.05 and ####p < 0.0001 compared with the PCOS group. The result is representative of three biological replicates. [file 3050487.f1.pdf]
